# Rearing behaviour in the mouse behavioural pattern monitor distinguishes the effects of psychedelics from those of lisuride and TBG

**DOI:** 10.3389/fphar.2023.1021729

**Published:** 2023-02-16

**Authors:** Yahong Chen, Junhong Liu, Yishan Yao, Haitao Yan, Ruibin Su

**Affiliations:** State Key Laboratory of Toxicology and Medical Countermeasures, Beijing Key Laboratory of Neuropsychopharmacology, Beijing Institute of Pharmacology and Toxicology, Beijing, China

**Keywords:** psychedelics, lisuride, TBG, 5-HT2A receptor, locomotor activity, exploratory rearing, jump

## Abstract

Psychedelics alter consciousness and may have potential for drug development. As psychedelics are likely therapeutically active, it is important to study their effects and mechanisms using preclinical models. Here, we examined the effects of phenylalkylamine and indoleamine psychedelics on locomotor activity and exploratory behaviour using the mouse Behavioural Pattern Monitor (BPM). DOM, mescaline, and psilocin reduced locomotor activity at high doses and influenced rearings, an exploratory behaviour, in a characteristic inverted U-shaped dose-response function. Pretreatment with the selective 5-HT_2A_ antagonist M100907 reversed the drug-induced alterations in locomotor activity, rearings, and jumps after systemic administration of DOM at low doses. However, holepoking at the full range of doses tested was not blocked by M100907. Administration of the hallucinogenic 5-HT_2A_ agonist 25CN-NBOH induced striking similarities in response to that to psychedelics; these alterations were significantly diminished by M100907, whereas the putatively non-hallucinogenic 5-HT_2A_ agonist TBG did not affect locomotor activity, rearings, or jumps at the most effective doses. The nonhallucinogenic 5-HT_2A_ agonist lisuride failed to increase rearing. The results of these experiments provide strong evidence that DOM-elicited increases in rearing are due to mediation by the 5-HT_2A_ receptor. Finally, discriminant analysis was able to distinguish all four psychedelics from lisuride and TBG based on behavioural performance alone. Thus, increased rearing in mice could provide additional evidence of behavioural differences between hallucinogenic and nonhallucinogenic 5-HT_2A_ agonists.

## Introduction

Psychedelics are defined as agents that are capable of altering cognition, perception, and mood without causing impairment to memory, intellectual function, or dependence, and that are associated with minimal adverse effects on the autonomic system (Hollister, 1964). Emerging clinical evidence indicates that psychedelics may prove useful as pharmacotherapy for many neuropsychiatric disorders (Chi and Gold, 2020). For example, recently completed phase II clinical trials showed that psilocybin is a viable alternative to current antidepressant medications (Ross et al., 2016; Carhart-Harris et al., 2018). However, despite the effectiveness of psychedelics in treating neuropsychiatric diseases, major hurdles of this research include the burden of their unique hallucinogenic effects.

Substantial evidence from animal and human studies (Fiorella et al., 1995a; Vollenweider et al., 1998; González-Maeso et al., 2003) has demonstrated that the characteristic effects of psychedelics are mediated by interactions with 5-HT_2A_ receptors (Kometer et al., 2013; Barrett et al., 2018; Preller et al., 2018; Nutt et al., 2020). However, one unresolved paradox is that only some 5-HT_2A_ agonists exhibit hallucinogenic activity, while structurally related agonists with similar affinities and activity lack such psychoactive effects. Indeed, the putatively nonhallucinogenic compound tabernanthalog (TBG), an analogue of the psychedelic 5-methoxy-N,N-dimethyltryptamine (5-MeO-DMT), was found not to induce the head-twitch response (HTR) in mice, a rodent behavioural proxy for the effects of psychedelics (Lu et al., 2021). Recently, the 5-HT_2A_ receptor antagonist ketanserin has been shown to block the effect of TBG on neural plasticity as well as its antidepressant effects (Cameron et al., 2021). As ketanserin blocks the therapeutic effects of TBG and has only weak to modest affinities for 5-HT_1B_ and α_2A_ adrenergic receptors (Peters and Olson, 2021), TBG may exert its effects on behaviour by activating 5-HT_2_ receptors. Furthermore, the ergoline derivative lisuride is a structural analogue of LSD and acts as a weak 5-HT_2A_ partial agonist (Newman-Tancredi et al., 2002). Despite its high affinity for 5-HT_2A_ receptors, lisuride does not exert a hallucinogenic effect in humans at acute doses of up to 400 μg (Herrmann et al., 1977; Verde et al., 1980; Raffaelli et al., 1983; Benes et al., 2006).

Many studies have compared the effects of hallucinogenic and nonhallucinogenic 5-HT_2A_ agonists to detect neurochemical differences that explain the ineffectiveness of the latter. Thus, the effects of lisuride on several behavioural patterns of animals known to be sensitive to the effects of psychedelics have been examined. High doses of lisuride (3.2 mg/kg) failed to induce a HTR in mice (Halberstadt and Geyer, 2013); in contrast, DOM induced a HTR even at very low doses (0.3 mg/kg) (Adam et al., 2020). Prepulse inhibition of acoustic startle response (PPI) is a cross-species phenomenon that can be assessed in humans and animals using similar testing procedures. Lisuride and LSD interfere with the PPI through different receptor mechanisms, suggesting that the PPI is a useful tool for comparing hallucinogenic and nonhallucinogenic 5-HT_2A_ agonists (Halberstadt and Geyer, 2010). Drug discrimination is a paradigm that can be used in multiple species and has a high pharmacological specificity to distinguish compounds with different mechanisms of action. However, regarding the use of this paradigm, there is a debate in the literature as to the extent to which the stimulatory effects of LSD, DOI, and DOM extend to lisuride, with studies reporting that lisuride partially or completely replaces those training drugs (Holohean et al., 1982; White and Appel, 1982; Glennon and Hauck, 1985; Fiorella et al., 1995b; Marona-Lewicka et al., 2002).

The BPM model has been widely used to study the effects of psychedelics on rodents, providing a qualitative and quantitative assessment of unconditioned locomotor and exploratory behaviours with clear conceptual relevance to the phenomenon of human hallucinogens, and thus has considerable construct validity (Adams and Geyer, 1985; Wing et al., 1990; Krebs-Thomson et al., 1998). In particular, this approach has proven beneficial in comparing the effects of different classes of psychostimulants that may generate comparable increases in locomotor activity at certain doses, but with dramatic qualitative differences in behaviour. Unlike the HTR paradigm, similar BPM models have been developed for humans, enabling comparative studies across species (Perry et al., 2009).

Considering these diverse findings, we tested the effects of hallucinogenic and nonhallucinogenic 5-HT_2A_ agonists on locomotor and exploratory behaviour using the mouse BPM. Additional experiments were conducted to determine the receptor mechanism underlying the impact of the phenylalkylamine psychedelic 2,5-dimethoxy-4-methylamphetamine (DOM) on locomotor activity and exploratory behaviour.

## Materials and methods

### Animals

For all experiments, male mice of the C57BL/6 strain weighing between 18 and 22 g were obtained from SPF Biotechnology Co., Ltd. (certificate no. SCXK 2019-0010, Beijing, China) and allowed to acclimate to the vivarium for at least 1 week after arrival. The male C57BL/6 mice were housed in cages with corncob bedding under a 12 h/12 h light/dark cycle (lights on at 8:00 a.m.), 60% ± 5% humidity, and a temperature of 23 ± 1^°^C with free access to water and food provided at the top of the cage. The experimental animals were acclimated to the experimental environment for 3 days before behavioural testing. Animal care and all experimental protocols were conducted in compliance with the Ethics Committee and Institutional Animal Care and Use Committee of Beijing Institute of Pharmacology and Toxicology, Beijing, China (IACUC of AMMS-06-2017-001).

### Drugs

The drugs used were N-(2-hydroxybenzyl)-2,5-dimethoxy-4-cyanophenylethylamine (25CN-NBOH; Axon Medchem BV, Groningen, Netherlands) hydrochloride; (R)-(+)-α-(2,3-dimethoxyphenyl)-1-[2-(4-fluorophenyl)ethyl]-4-piperidinemethanol (M100907); lisuride maleate (Topscience, Shanghai, China); tabernanthalog (TBG) hydrochloride, psilocin hydrochloride, 2,5-dimethoxy-4-methylamphetamine (DOM) hydrochloride, and mescaline hydrochloride (donated by Dr. Yao Yishan, Beijing, China). M100907, lisuride, and 25CN-NBOH were dissolved in isotonic saline containing 1% DMSO. The remaining drugs were all dissolved in isotonic saline. All drugs were administered intraperitoneally at a volume of 10 mL per 1 kg mouse bodyweight.

### Apparatus

**FIGURE 1:**
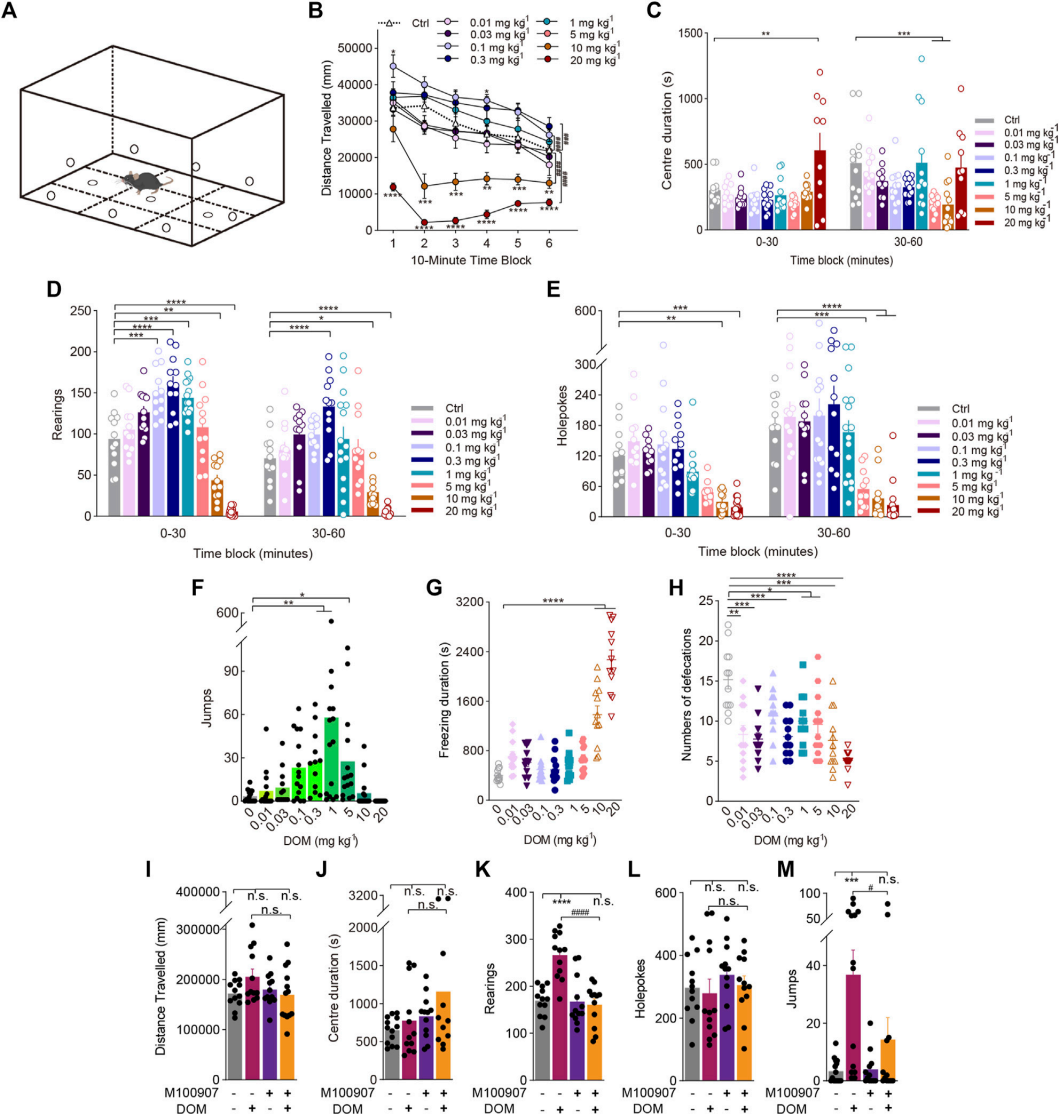
Behavioural changes in locomotor and exploratory behaviour of mice induced by the phenylalkylamine psychedelic DOM. **(A)** Schematic of the mouse BPM. Effect of DOM on **(B,I)** distance travelled (in mm), **(C,J)** centre duration (in seconds), **(D,K)** the total number of rearing behaviours (*rearings*), **(E,L)** the total number of hole explorations (*holepokes*), **(F,M)** the total number of jumps (*jumps*), **(G)** freezing duration (in seconds), and **(H)** the total number of defecations. Data are shown as the means ± SEM for consecutive 10-min **(B)** and 30-min intervals **(C,D,E)**. *n* = 12–15 mice per group. Statistical tests included two-way repeated-measures ANOVA comparing treatment groups to the control group at each timepoint **(B)** as well as, one-way ANOVA **(G,H)**, and two-way **(B–E)** ANOVA followed by Dunnett’s multiple comparisons test and Kruskal-Wallis H test with Dunn’s multiple comparisons test **(F)**. ^*^ *p* < 0.05, ^**^ *p* < 0.01, ^***^ *p* < 0.001, ^****^ *p* < 0.0001, and ^###^ *p* < 0.001, ^####^ *p* < 0.0001, significantly different from the control group **(A–H)**. **(I–M)** Effect of pretreatment with the 5-HT_2A_ antagonist M100907 (1 mg/kg DOM +0.01 mg/kg M100907). *n* = 12–15 mice per group. Data were analysed by two-way ANOVA followed by Bonferroni’s multiple comparison test. Data are presented as group means ± SEM. ^***^ *p* < 0.001, ^****^ *p* < 0.0001, significantly different from the control group; ^#^ *p* < 0.05, ^####^ *p* < 0.0001, significantly different from mice given DOM alone; n. s., not significant.

As previously mentioned, locomotor and exploratory behaviour were tested in the mouse BPM (Risbrough et al., 2006; van Enkhuizen et al., 2013) (Beijing Zhongshidichuang Science and Technology Development Co., Ltd, Beijing, China). The mouse BPM contains eight holes (1.25 cm in diameter and 1.90 cm above the floor) in the walls of the Plexiglas chamber (30.5 cm × 61 cm × 38 cm) and three holes in the floor (Figure 1A). Each hole has an infrared beam to detect entries (e.g., holepokes). A grid of 12 × 24 infrared beams, located 1 cm above the floor, records the position of the mouse every 0.1 s, allowing the calculation of movements from one of the nine defined areas to another. The second pair of 16 infrared beams located 2.5 cm above the floor monitor the number of rearing behaviours. At the start, each mouse was placed in the centre of the apparatus. The bottom of the chamber is divided into 16 small squares. The four small squares in the centre were defined as the central area. The mice were allowed to explore the mouse BPM for 60 min. In each chamber, external lighting and noise were minimized and recording was performed under internal white light (350 lx in the centre of the four chambers and 92 lx in the corners). The chambers were cleaned between testing sessions with 75% ethanol.

### Procedures

Ten minutes after administration of DOM, mescaline, psilocin, 25CN-NBOH, lisuride, or TBG; or 30 min after administration of M100907, the animals were placed in the mouse BPM. The behaviours of mice were recorded in the chamber for 60 min.

### Data analysis

Horizontal locomotor activity was measured in terms of distance travelled. The number of rearing and holepoking behaviours were used as an index of exploratory behaviour. Temporal resolutions of 10, 30, and 60 min were used to analyse the BPM data of mice. Prism 8.3.0 (Graphpad, San Diego, CA, United States) was utilized for statistical analysis. The results are presented as the sample means ± SEM unless otherwise stated. Differences among multiple groups were assessed by the Kruskal-Wallis H test, one-way analysis of variance (ANOVA), or two-way ANOVA for multifactorial analyses. As appropriate, Bonferroni, and Dunnett’s *post hoc* tests were employed to determine group differences. A *p*-value <0.05 was considered statistically significant for all statistical measures. Data collection and analysis were conducted without knowledge of the experimental circumstances. All mice were arbitrarily divided into distinct treatment groups.

Discriminant analysis is a multivariate statistical technique (based on martingale distance and stepwise-forward procedures); this analysis was applied to 1) distinguish groups that had received hallucinogenics from those that had received nonhallucinogenics according to their behavioural characteristics; 2) identify differential variables that significantly affected group differences; 3) determine the best way to distinguish groups; and 4) identify unclassified individuals (Vekovischeva et al., 2013). Statistical analyses were performed using the predictive analysis software IBM SPSS 20.0. The α level of all behavioural characteristic values discussed in this study was 0.05.

## Results

### Effect of DOM on locomotor and exploratory behaviour

Figure 1B depicts the influence of different dosages of DOM on locomotor activity, as measured by distance travelled, across multiple 10-min intervals in the 1-h test period. DOM influenced locomotor activity in a bell-shaped dose-response curve, with low and medium doses leading to increased later locomotor activity, while high doses (≥10 mg/kg) resulted in decreased activity at the start of the trial [Treatment: F_(8, 105)_ = 30.42, *p* < 0.0001; Treatment × Block: F_(40, 525)_ = 2.793, *p* < 0.0001]. DOM at doses of 0.1 (*p* < 0.0001) and 0.3 mg/kg (*p* = 0.0008) increased the distance travelled relative to that after administering the vehicle. Post hoc analyses revealed that 0.1 mg/kg DOM induced significantly higher locomotor activity throughout the first (*p =* 0.0336) and fourth 10-min time blocks (*p =* 0.0143), while 1 mg/kg DOM induced an increasing trend but no significant difference in locomotor activity (*p =* 0.3335). Interestingly, however, during the initial 10-min time block, the trend of distance travelled after the high dose (10 mg/kg) of DOM was not significantly different compared to that after the vehicle (*p =* 0.5979), whereas 20 mg/kg DOM dramatically reduced the distance travelled throughout the whole test (*p* < 0.0001).

DOM substantially influenced holepokes, rearings, and jumps. DOM decreased holepoking behaviour in a dose dependently manner throughout the whole 60-min session [Treatment: F_(8, 107)_ = 16.25, *p* < 0.0001; Treatment × Block: F_(8, 107)_ = 2.596, *p* = 0.0124; Figure 1E]. There was a trend towards an interaction between treatment and time block in rearing behaviour. Consistent with the effects of DOM on locomotor activity, DOM influenced rearing [Treatment: F_(8, 104)_ = 34.33, *p* < 0.0001; Treatment × Block: F_(8, 103)_ = 3.101, *p* = 0.0035; Figure 1D] and jumps (χ^2^ = 61.15, *p* < 0.0001; Figure 1F), with an increase in exploratory activity at low to moderate doses and a decrease at higher doses, following a bell-shaped dose-response function. Notably, 1 mg/kg DOM considerably increased rearings (*p* = 0.0006) and jumps (*p* = 0.0021), indicating that while DOM was able to influence exploratory behaviour, a low dose was not sufficient to markedly alter locomotor activity or patterns. Conversely, 10 (*p* = 0.0013) and 20 mg/kg DOM reduced rearing (*p* < 0.0001).

According to preliminary HTR studies (Adam et al., 2020), 1 mg/kg DOM exerted significantly greater effects than other doses (data not shown). Therefore, the dose used in subsequent behavioural experiments was 1 mg/kg.

To determine whether mice exhibited anxiety-like behaviour following the administration of DOM, the amount of time spent in the central area of the mouse BPM was observed. A dose of 1 mg/kg DOM was insufficient to distinguish centre duration from that exhibited by the vehicle group, but 20 mg/kg DOM induced a significant increase in centre duration [Treatment: F_(8, 96)_ = 4.816, *p* < 0.0001; Treatment × Block: F_(8, 96)_ = 4.071, *p* = 0.0003; Figure 1C]. To clarify whether DOM-induced freezing behaviour reflected fear-induced freezing, the duration of freezing during the test period was measured. Only 10 and 20 mg/kg DOM increased freezing duration significantly compared to that in the vehicle group (F_(8, 99)_ = 46.58, *p* < 0.0001). Other dose groups were not significantly different (Figure 1G). Studies in animals and humans have shown that the autonomic nervous system undergoes rapid changes in response to fear. At the dose range tested (0.01–20 mg/kg), DOM significantly reduced defecation in mice (F_(8, 99)_ = 8.948, *p* < 0.0001; Figure 1H).

To confirm that the increase in locomotor activity, rearing, and jumping behaviour induced by DOM was mediated by the 5-HT_2A_ receptor, we examined whether the effect of 1 mg/kg DOM was attenuated by pretreatment with the selective 5-HT_2A_ antagonist M100907. The results in Figures 1I, J, L illustrate that 1 mg/kg DOM did not significantly influence the distance travelled (F_(1, 12)_ = 0.9143, *p* = 0.3578), centre duration (F_(1, 12)_ = 1.878, *p* = 0.1956) or the number of holepokes (F_(1, 11)_ = 0.6446, *p* = 0.4391), nor did it influence the interaction of pretreatment with treatment [Treatment × Pretreatment: F_(1, 9)distance travelled_ = 3.097, *p* = 0.1123; F_(1, 8)centre duration_ = 0.4608, *p* = 0.5164; F_(1, 11)holepokes_ = 0.05339, *p* = 0.8215]. As expected, 1 mg/kg DOM increased rearing (F_(1, 11)_ = 16.64, *p* = 0.0018), and jumps (F_(1, 49)_ = 14.29, *p* = 0.0004). The ability of DOM to increase rearing was attenuated by M100907 [Treatment × Pretreatment: F_(1, 11)_ = 30.31, *p* = 0.0002], and this finding was supported by a *post hoc* test (*p* < 0.0001; Figure 1K). Pretreatment with M100907 also blocked DOM-induced increases in jumps [Treatment × Pretreatment: F_(1, 49)_ = 3.983, *p* = 0.0004], as confirmed by *post hoc* tests (*p* = 0.0464; Figure 1M).

### Effect of mescaline on locomotor and exploratory behaviour

**FIGURE 2:**
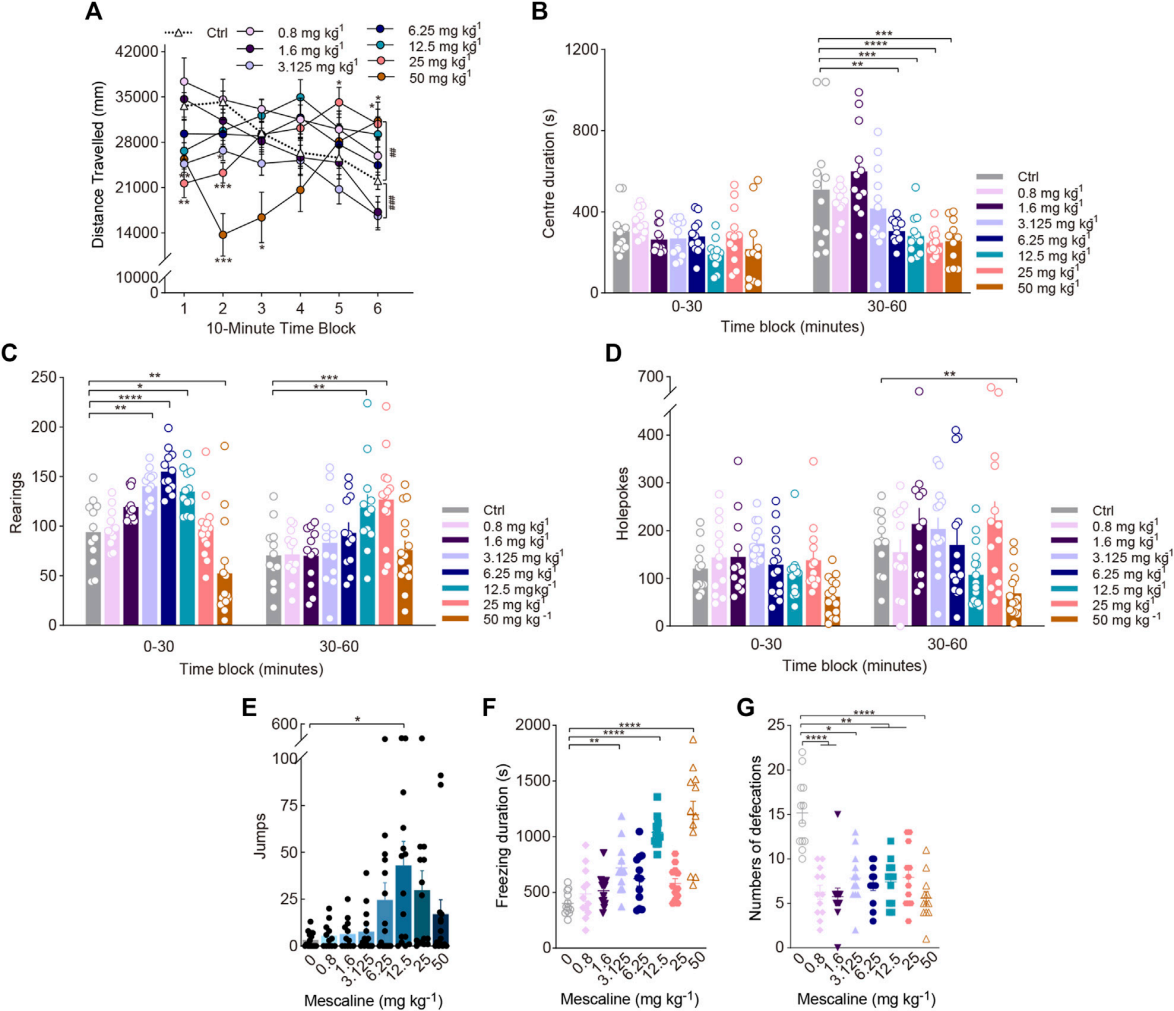
Behavioural changes in locomotor and exploratory behaviour of mice induced by the phenylalkylamine psychedelic mescaline. The effect of mescaline on **(A)** distance travelled (in mm), **(B)** centre duration (in seconds), **(C)** the total number of rearing behaviours (*rearings*), **(D)** the total number of hole explorations (*holepokes*), **(E)** the total number of jumps (*jumps*), **(F)** freezing duration (in seconds), and **(G)** the total number of defecations. Data are shown as the means ± SEM for consecutive 10-min **(A)** and 30-min intervals **(B–D)**. *n* = 12 mice per group. Statistical tests included two-way repeated measures **(A)** as well as, one-way **(F)**, and two-way **(A–D)** ANOVA followed by Dunnett’s multiple comparisons tests. Kruskal-Wallis H with Dunn’s multiple comparisons tests **(E,G)** comparing treatment groups to the control group. ^*^ *p* < 0.05, ^**^ *p* < 0.01, ^***^ *p* < 0.001, ^****^ *p* < 0.0001, ^##^ *p* < 0.01, and ^###^ *p* < 0.001.

To further determine whether other phenylalkylamine psychedelics exhibited similar effects to DOM in terms of increases in rearing, we tested mescaline. Mescaline exhibited the same dose- and time-dependency with respect to its effects on locomotor activity. Increases in the mescaline dose (3.125 and 25 mg/kg) markedly decreased the distance travelled throughout the first 20 min of the test [Treatment: F_(7, 100)_ = 3.295, *p* = 0.0034; Treatment × Block: F_(35, 500)_ = 7.215, *p* < 0.0001; Figure 2A](*p* < 0.01, *p* < 0.01), and 50 mg/kg mescaline markedly decreased the distance travelled midway through the test (*p* < 0.0001, *p* < 0.05). Moreover, the last 10-min of the 1-h test session was characterized by a pronounced increase in locomotor activity in response to mescaline at doses between 25 and 50 mg/kg (*p* < 0.05, *p* < 0.05).

Similar to a previous study, there was a significant effect of drug treatment on rearing as well as a treatment × time interaction. Rearing exhibited biphasic effects, with doses of 3.125 (*p* = 0.0052), 6.25 (*p* < 0.0001), and 12.5 mg/kg (*p* = 0.0183) increasing rearing in the first 30-min time block, and a dose of 50 mg/kg decreasing rearing [Treatment: F_(7, 93)_ = 7.770, *p* < 0.0001; Treatment × Block: F_(7, 93)_ = 14.12, *p* < 0.0001; Figure 2C]. There were significant effects of mescaline on holepoking. Pairwise comparisons indicated that administration of 50 mg/kg mescaline reduced holepoking throughout the last 30 min of the test (F_(7, 101)_ = 4.838, *p* < 0.0001; Figure 2D). Additionally, we found that 12.5 mg/kg mescaline substantially increased the number of jumps (χ^2^ = 14.82, *p* = 0.0384; Figure 2E). Despite a significant increase in freezing duration induced by 3.125 and 12.5 mg/kg mescaline (F_(7, 88)_ = 18.80, *p* < 0.0001; Figure 2F), defecation was markedly reduced by 0.8–50 mg/kg mescaline (χ^2^ = 34.73, *p* < 0.0001; Figure 2G) and there was no obvious difference in the centre duration during the first 30-min of testing (Figure 2B).

### Effect of psilocin on locomotor and exploratory behaviour

**FIGURE 3:**
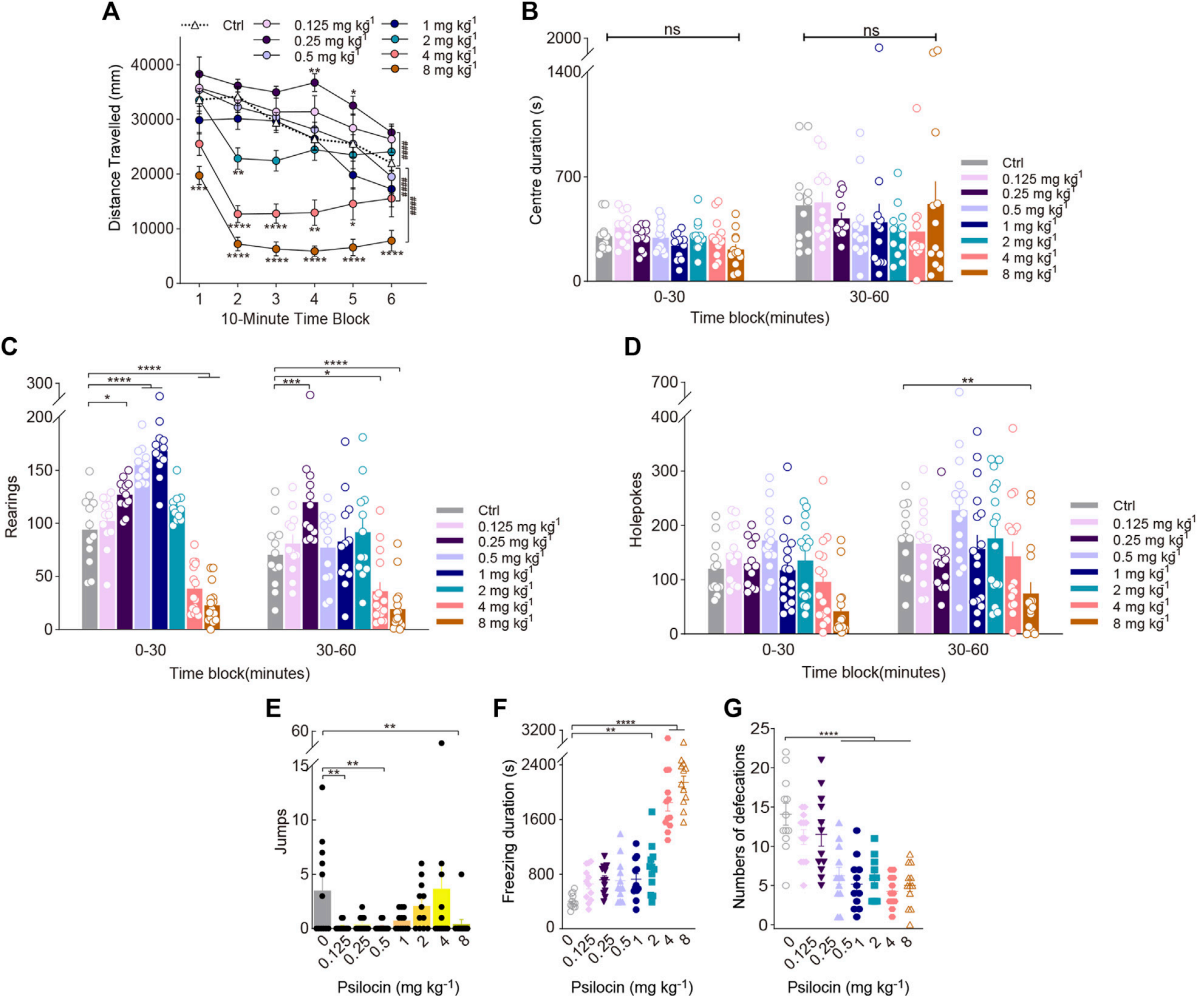
Behavioural changes in locomotor activity and exploratory behaviour of mice induced by the indoleamine psychedelic psilocin. The effect of psilocin on **(A)** distance travelled (in mm), **(B)** centre duration (in seconds), **(C)** the total number of rearing behaviours (*rearings*), **(D)** the total number of hole explorations (*holepokes*), **(E)** the total number of jumps (*jumps*), **(F)** freezing duration (in seconds), and **(G)** the total number of defecations. Data are shown as the means ± SEM for consecutive 10-min **(A)** and 30-min intervals **(B–D)**. *n* = 12 mice per group. Statistical tests included two-way repeated measures **(A)**, one-way **(F,G)**, and two-way **(A–D)** ANOVA followed by Dunnett’s multiple comparisons tests. Kruskal-Wallis H test with Dunn’s multiple comparisons tests **(E)** comparing treatment groups to the control group. *p < 0.05, **p < 0.01, ***p < 0.001, ****p < 0.0001, and ^####^ *p* < 0.0001.

To further determine whether indoleamine psychedelics exhibit similar effects to DOM in terms of their ability to increase rearing, we tested psilocin. As shown in Figure 3A, psilocin decreased locomotor activity, as measured by the distance travelled (F_(7, 106)_ = 27.22, *p* < 0.0001). The highest dose tested (8 mg/kg psilocin) was demonstrated to be the most effective. There was also an interaction between treatment and time (Treatment × Block: F_(35, 530)_ = 3.654, *p* < 0.0001). Post hoc analyses revealed that psilocin at the dose of 2 mg/kg significantly decreased the distance travelled during block 2 (*p* = 0.0011), and psilocin at a dose of 4 mg/kg reduced the distance travelled during blocks 2-5 (*p* < 0.0001), whereas psilocin at a dose of 8 mg/kg reduced the distance travelled for a longer duration (*p* < 0.0001). Conversely, psilocin significantly increased the distance travelled during blocks 4**–**5 when administered at a dose of 0.25 mg/kg (*p* < 0.01 and *p* < 0.05, respectively).

Post hoc analyses showed that psilocin at 0.25 (*p* = 0.0363), 0.5 (*p* < 0.0001), and 1 mg/kg (*p* < 0.0001) significantly increased rearing during the first 30-min block and doses of 4 (*p* < 0.0001) and 8 mg/kg psilocin (*p* < 0.0001) gradually decreased rearing, resulting in a treatment × time interaction (Treatment × Block: F_(7, 95)_ = 9.577, *p* < 0.0001; Figure 3C). There were significant effects of psilocin on holepoking behaviour. Pairwise comparisons suggested that psilocin administered at 8 mg/kg markedly reduced holepoking throughout the last 30 min of the test (F_(7,107)_ = 4.964, *p* < 0.0001; Figure 3D). Likewise, only the high doses of psilocin (e.g., 2, 4, and 8 mg/kg) resulted in substantial increases in freezing duration (F_(7, 88)_ = 52.54, *p* < 0.0001; Figure 3F); 1 mg/kg psilocin did not statistically impact locomotor activity. Moreover, defecation at the doses tested (0.5–8 mg/kg) was significantly reduced (F_(7, 88)_ = 13.14, *p* < 0.0001; Figure 3G). There was no effect of treatment with psilocin at the dose tested on centre duration (Figure 3B). It should be noted that there was a tendency for psilocin treatment at 4 mg/kg to produce an increase in jumping during the test session, but *post hoc* analyses did not confirm this effect (Figure 3E).

### Effect of 25CN-NBOH on locomotor and exploratory behaviour

**FIGURE 4:**
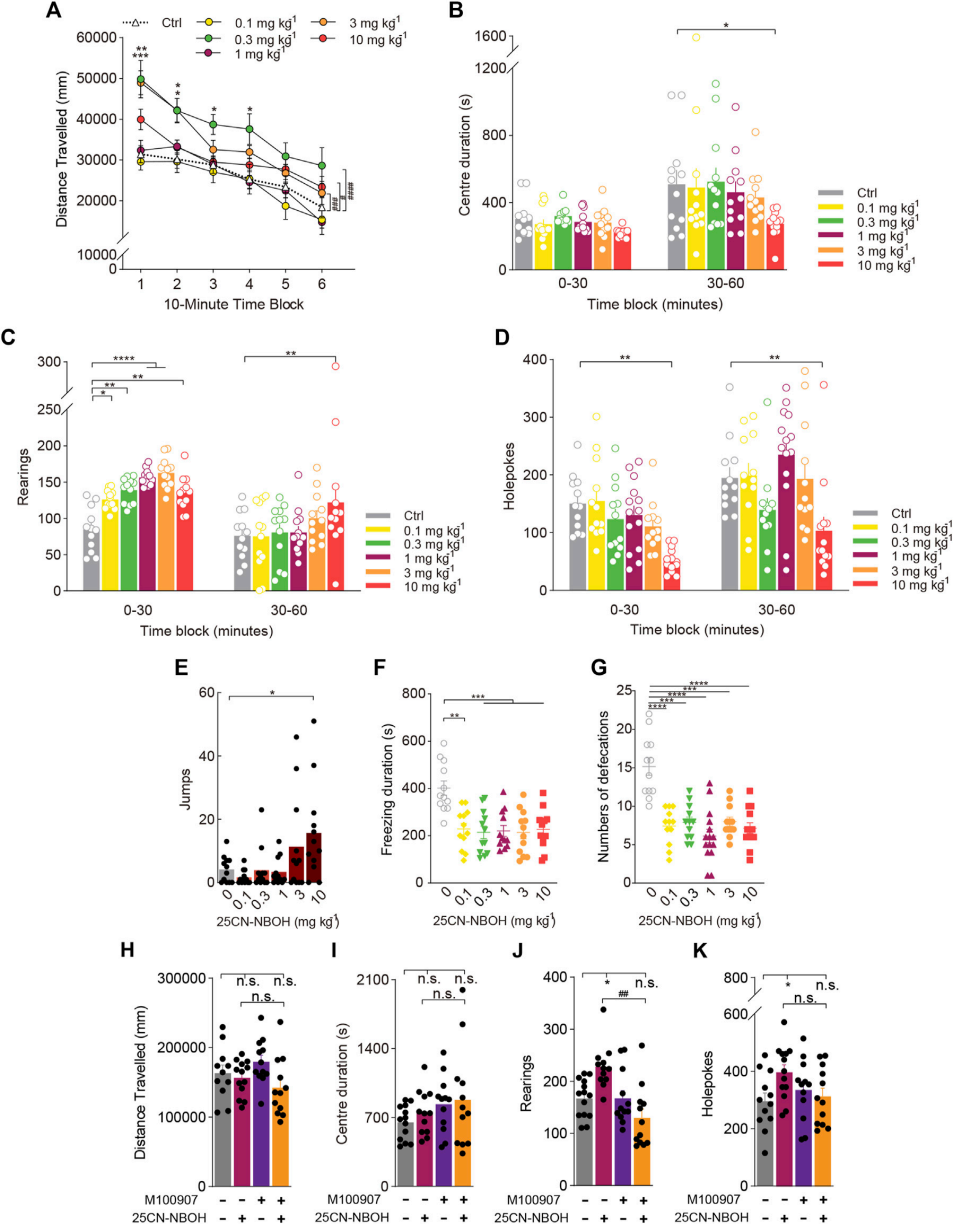
Behavioural changes in locomotor and exploratory behaviour of mice induced by the hallucinogenic 5-HT_2A_-selective agonist 25CN-NBOH. The effect of 25CN-NBOH on **(A,H)** distance travelled (in mm), **(B,I)** centre duration (in seconds), **(C,J)** the total number of rearing behaviours (*rearings*), **(D,K)** the total number of hole explorations (*holepokes*), **(E)** the total number of jumps (*jumps*), **(F)** freezing duration (in seconds), and **(G)** the total number of defecations. Data are shown as the means ± SEM for consecutive 10-min **(A)** and 30-min intervals **(B–D)**. *n* = 12 mice per group. Statistical tests included two-way repeated measures **(A)**, one-way **(E–G)**, and two-way **(A–D)** ANOVA followed by Dunnett’s multiple comparisons tests comparing treatment groups to the vehicle control group. *p < 0.05, **p < 0.01, ***p < 0.001, ****p < 0.0001, and ^#^ *p* < 0.05, ^###^ *p* < 0.001, ^####^ *p* < 0.0001. **(H–K)** Effect of pretreatment with the 5-HT_2A_ antagonist M100907 (1 mg/kg 25CN-NBOH +0.01 mg/kg M100907). *n* = 12 mice per group. Data were analysed by two-way ANOVA followed by Bonferroni’s multiple comparison test. Data are presented as group means ± SEM. *p < 0.05, significantly different from the control group; ^##^ *p* < 0.01, significantly different from mice given 25CN-NBOH alone; n. s., not significant.

To further verify whether DOM-induced increases in rearing behaviour were mediated by the 5-HT_2A_ receptor, we compared the effects of DOM with those of the classic hallucinogenic 5-HT_2A_ agonist 25CN-NBOH. Treatment with 0.3 (*p* < 0.0001), 3 (*p* = 0.0002), and 10 (*p* = 0.0241) mg/kg 25CN-NBOH increased the distance travelled, and there was an interaction between 25CN-NBOH treatment and time [Treatment: F_(5, 68)_ = 6.676, *p* < 0.0001; Treatment × Block: F_(25, 340)_ = 2.015, *p* = 0.0032; Figure 4A]. Based on *post hoc* analyses, we found that 0.3 and 3 mg/kg 25CN-NBOH markedly increased the distance travelled throughout the first 40 min (*p* < 0.01) and the first 20 min of the test (*p* < 0.01), respectively. In contrast, 1 mg/kg 25CN-NBOH did not significantly alter the distance travelled during the entire test session.

As shown in Figure 4C, 25CN-NBOH [Treatment: F_(5, 67)_ = 5.758, *p* = 0.0002; Treatment × Block: F_(5, 66)_ = 4.758, *p* = 0.0009] increased rearing behaviour in a dose-dependent manner. It would appear that 25CN-NBOH has a similar dose-response curve to that of DOM. There was a trend towards an interaction of 25CN-NBOH treatment and time block for holepokes [Treatment: F_(5, 69)_ = 6.004, *p* = 0.0001; Treatment × Block: F_(5, 69)_ = 2.221, *p* = 0.0618], and *post hoc* analysis confirmed this effect for any specific 30-min time block (*p* < 0.01, *p* < 0.01; Figure 4D). Notably, 1 mg/kg 25CN-NBOH promoted rearing without affecting spontaneous activity but not holepoking (*p* < 0.0001). Furthermore, the results show that compared to the vehicle group, 10 mg/kg 25CN-NBOH considerably increased jumping (F_(5, 66)_ = 2.221, *p* = 0.0033), whereas 1 mg/kg 25CN-NBOH did not substantially impact jumping (Figure 4E). In addition, we found that 25CN-NBOH did not alter the centre duration of mice at doses of 0.1–10 mg/kg (F_(5, 66)_ = 0.7473, NS; Figure 4B), but greatly reduced freezing duration (F_(5, 66)_ = 8.177, *p* < 0.0001; Figure 4F) and defecation (F_(5, 70)_ = 16.18, *p* < 0.0001; Figure 4G).

To determine whether the increase in rearing induced by 25CN-NBOH was mediated by the 5-HT_2A_ receptor, we compared the effect of 1 mg/kg 25CN-NBOH in mice pretreated with the 5-HT_2A_ antagonist M100907. Treatment with 1 mg/kg 25CN-NBOH did not significantly alter the distance travelled and centre duration, and there was no interaction between M100907 pretreatment and 25CN-NBOH treatment (F_(1, 8)distance travelled_ = 2.766, NS; F_(1, 9)center duration_ = 0.1504, NS; Figures 4H, I). Similar to DOM, pretreatment with M100907 attenuated the increase in rearing behaviour induced by 1 mg/kg 25CN-NBOH [Treatment: F_(1, 13)_ = 14.33, *p* = 0.0023; Treatment × Pretreatment: F_(1, 7)_ = 14.91, *p* = 0.0062; Figure 4J]. There was also a pretreatment × treatment interaction for holepoking (F_(1, 9)_ = 6.844, *p* = 0.0280; Figure 4K), but *post hoc* analyses demonstrated that M100907 failed to significantly attenuate the effect of 25CN-NBOH on holepoking.

### Effect of lisuride on locomotor and exploratory behaviour

**FIGURE 5:**
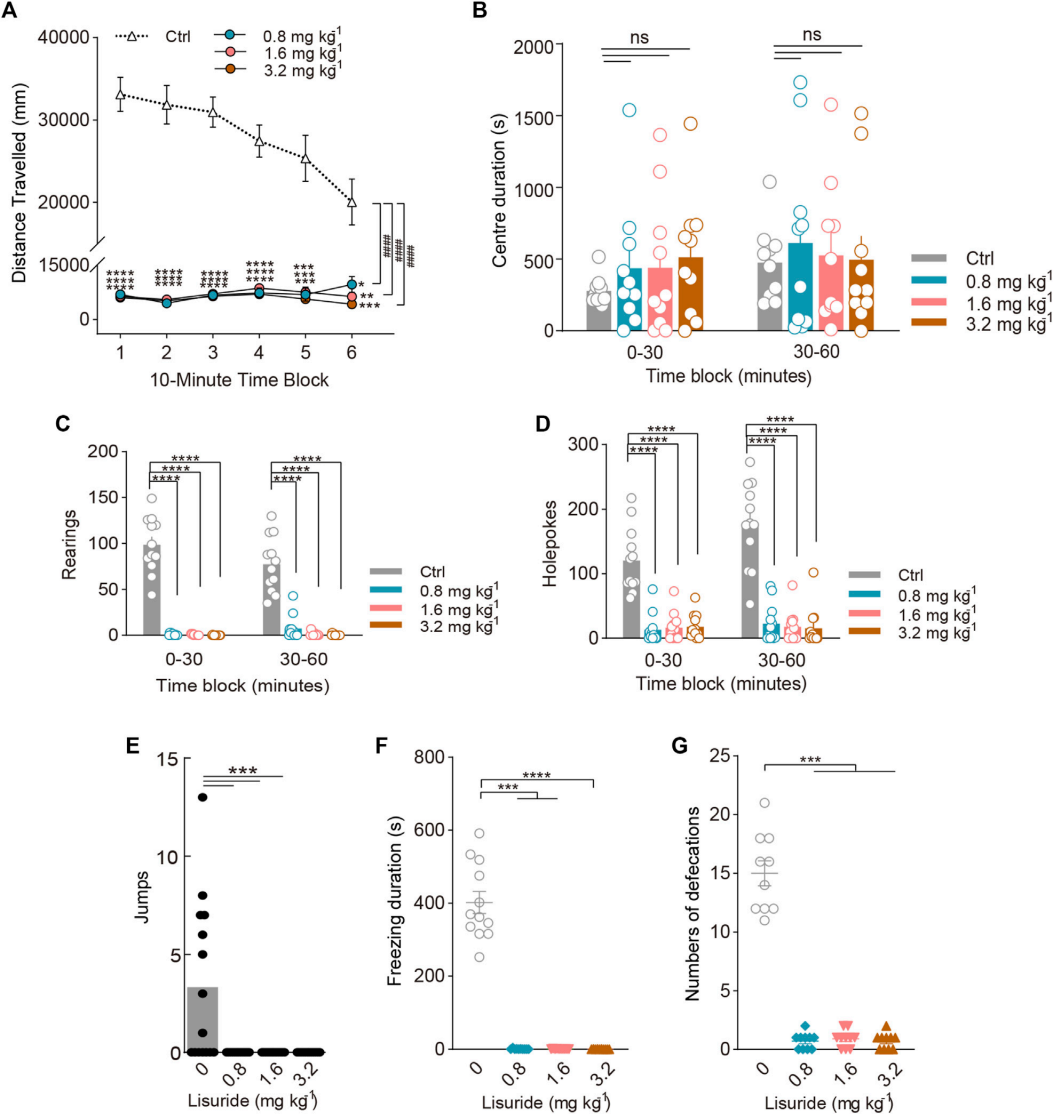
Behavioural changes in locomotor and exploratory behaviour of mice induced by the nonhallucinogenic 5-HT_2A_-selective agonist lisuride. The effect of lisuride on **(A)** distance travelled (in mm), **(B)** centre duration (in seconds), **(C)** the total number of rearing behaviours (*rearings*), **(D)** the total number of hole explorations (*holepokes*), **(E)** the total number of jumps (*jumps*), **(F)** freezing duration (in seconds), and **(G)** the total number of defecations. Data are shown as the means ± SEM for consecutive 10-min **(A)** and 30-min intervals **(B–D)**. *n* = 12 mice per group. Statistical tests included two-way repeated measures **(A)**, and two-way **(A–D)** ANOVA followed by Dunnett’s multiple comparisons test. Kruskal-Wallis H test with Dunn’s multiple comparisons tests **(E–G)** comparing treatment groups to the control group. *p < 0.05, **p < 0.01, ***p < 0.001, ****p < 0.0001, and ^####^ *p* < 0.0001.

To determine whether the 5-HT_2A_-induced increase in rearing behaviour was specific to psychedelics, we examined the effects of the nonhallucinogenic 5-HT_2A_ agonist lisuride. There was a significant main effect of lisuride on the distance travelled during the whole test session [Treatment: F_(3, 36)_ = 83.94, *p* < 0.0001; Treatment × Block: F_(15, 180)_ = 8.581, *p* < 0.0001; Figure 5A], and *post hoc* analyses demonstrated that even 3.2 mg/kg lisuride significantly reduced the distance travelled (*p* < 0.0001). Rearing (F_(3, 44)_ = 105.2, *p* < 0.0001) and holepoking (F_(3, 44)_ = 54.22, *p* < 0.0001) were also reduced by lisuride throughout the 1-h session, resulting in interactions between treatment and block [Treatment × Block: F_(3, 44)rearing_ = 11.16, *p* < 0.0001; F_(3, 44)holepokes_ = 6.185, *p* = 0.0013; Figures 5C, D]. As shown in Figure 5E, mice treated with lisuride jumped significantly fewer times (χ^2^ = 22.12, *p* < 0.0001). Likewise, freezing duration (χ^2^ = 26.17, *p* < 0.0001; Figure 5F) and defecations (χ^2^ = 23.99, *p* < 0.0001; Figure 5G) were also reduced by lisuride in a dose-dependent manner. There was no interaction of lisuride treatment with block in terms of the centre duration (Treatment × Block: F_(3, 36)_ = 0.4702, NS; Figure 5B).

### Effect of TBG on locomotor and exploratory behaviour

**FIGURE 6:**
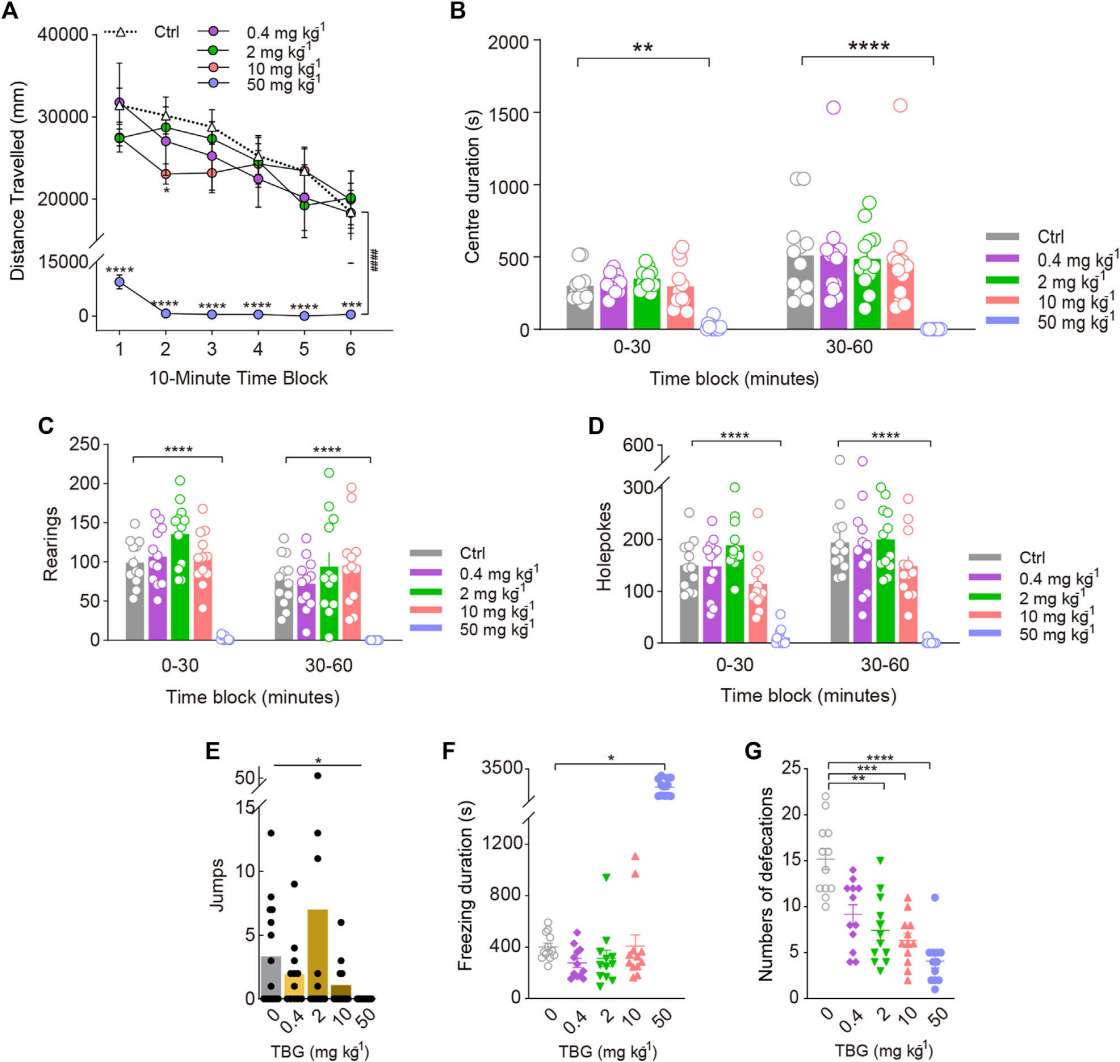
Behavioural changes in locomotor and exploratory behaviour of mice induced by the putatively nonhallucinogenic 5-HT_2A_-selective agonist TBG. The effect of TBG on **(A)** distance travelled (in mm), **(B)** centre duration (in seconds), **(C)** the total number of rearing behaviours (*rearings*), **(D)** the total number of hole explorations (*holepokes*), **(E)** the total number of jumps (*jumps*), **(F)** freezing duration (in seconds), and **(G)** the total number of defecations. Data are shown as the means ± SEM for consecutive 10-min **(A)** and 30-min intervals **(B–D)**. *n* = 12 mice per group. Statistical tests included two-way repeated measures **(A)**, and two-way **(A–D)** ANOVA followed by Dunnett’s multiple comparisons test. Kruskal-Wallis H test with Dunn’s multiple comparisons tests **(E–G)** comparing treatment groups to the control group. *p < 0.05, **p < 0.01, ***p < 0.001, ****p < 0.0001, and ^####^ *p* < 0.0001.

To further validate that the 5-HT_2A_-induced increase in rearing behaviour was specifically associated with hallucinogenic activity, we compared its effects with those of the putatively nonhallucinogenic 5-HT_2A_ agonist TBG. As expected, mice exposed to 0.4 and 2 mg/kg TBG did not exhibit any changes in the distance travelled. Post hoc analysis revealed that 10 mg/kg TBG markedly decreased the distance travelled throughout the first 20 min of the test [Treatment: F_(4, 55)_ = 20.44, *p* < 0.0001; Treatment × Block: F_(20, 275)_ = 1.962, *p* = 0.0092; Figure 6A] (*p* < 0.05), and TBG at the dose of 50 mg/kg significantly decreased the distance travelled throughout the entire test session [F_(5, 66)_ = 13.07, *p* < 0.0001].

There was a significant effect of TBG on holepoking [F_(4, 55)_ = 31.66, *p* < 0.0001; Figure 6D], rearing [Treatment: F_(4, 55)_ = 20.54, *p* < 0.0001; Treatment × Block: F_(4, 55)_ = 3.609, *p* = 0.0111; Figure 6C], and jumping (χ^2^ = 10.38, *p* = 0.0345). Contrary to the behavioural effects of the DOM, only the 50 mg/kg dose of TBG significantly reduced holepoking (*p* < 0.0001, *p* < 0.0001) and rearing (*p* < 0.0001, *p* < 0.0001) during all test sessions, as demonstrated by pairwise comparisons. Likewise, 50 mg/kg TBG contributed to a reduction in jumping (*p* = 0.0142; Figure 6E). Furthermore, no difference was observed between the effects of 0.4 and 10 mg/kg TBG on rearing, holepoking, and jumping in mice.

Additionally, high doses of TBG caused stereotypical behaviour, resulting in an increase in freezing duration (χ^2^ = 33.17, *p* < 0.0001; Figure 6F) and a decrease in centre duration (F_(4, 55)_ = 13.29, *p* < 0.0001; Figure 6B); at the dose tested (2–50 mg/kg), TBG was shown to significantly reduce defecation (χ^2^ = 31.54, *p* < 0.0001; Figure 6G). Nevertheless, there was no significant difference in freezing duration and centre duration between 0.4 and 10 mg/kg TBG.

### Discriminant analysis

**FIGURE 7:**
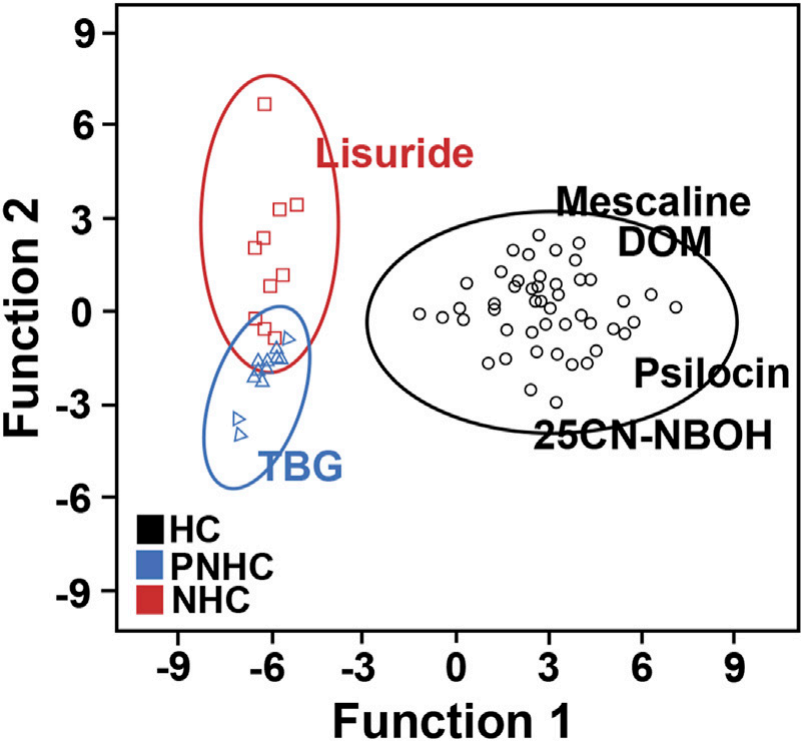
Graphical representation of the results from discriminant function analysis of all behavioural parameters. HC, hallucinogens; PNHC, putative nonhallucinogens; NHC, nonhallucinogens.

Discriminant analysis successfully distinguished hallucinogenic, putative nonhallucinogenic, and nonhallucinogenic compounds. The discriminant functions were constructed from the set of behavioural parameters. Standardized canonical discriminant function coefficients are illustrated in Figure 7. In addition, canonical discriminant Function 1 explained 94.8% of the variance with good statistical significance at *p* < 0.001, while canonical discriminant Function 2 explained only 5.2% of the variance, indicating that Function 1 explained essentially all of the variance. Discriminant functions built on behavioural parameters identified “Rearing,” “Holepoke,” “Center duration,” and “Freezing” as principal variables for Function 1. Symbols of the main principal behavioural elements are presented in Table 1.

**TABLE 1:** Discriminant function coefficients of the main behavioural elements that determined the behavioural function.

		Coefficients	
Behavioural categories	Behavioural elements	Function 1^a^	Function 2^b^
Defence	Freezing (Fz)	0.128	−0.466
Exploratory behaviour	Rearing (R)	0.926	−0.072
	Holepoke (Hp)	0.262	0.049
Other behaviours	Centre duration (Cd)	0.011	0.876

A linear discriminant function was constructed to distinguish between the behavioural characteristics induced by hallucinogenics compared to nonhallucinogenics.

^a^ Function 1 = Fz × (0.128) + R × (0.926) + Hp × (0.262) + Cd × (0.011).

^b^ Function 2 = Fz × (−0.466) + R × (−0.072) + Hp × (0.049) + Cd × (0.876).

## Discussion

It is well known that psychedelics exert their specific effects by activating 5-HT_2A_ receptors (Nichols, 2004; Halberstadt and Geyer, 2011). The phenylalkylamine hallucinogen DOI has been previously reported to increase the locomotor activity of C57BL/6J mice; this increase is ameliorated by deleting the 5-HT_2A_ receptor gene, indicating that this effect is mediated by the 5-HT_2A_ receptor (Halberstadt et al., 2009). In the current study, we compared the effects of DOM treatment with those of treatment with other phenylalkylamine- or indoleamine-based psychedelics on the locomotor and exploratory behaviours of C57BL/6J mice and determined the contribution of 5-HT_2A_ receptors to these behavioural effects. Similar to DOI, the phenylalkylamine psychedelics DOM and mescaline induced locomotor hyperactivity in mice. Notably, DOM exhibited a dose-response curve with an inverted U shape, with lower doses (0.1 mg/kg) resulting in increased locomotor activity and higher doses (≥10 mg/kg) causing a decrease in locomotor activity. Compared with phenylalkylamine psychedelics, the indoleamine psychedelic psilocin exhibited a distinct profile of effects, with reduced locomotor activity observed in the mouse BPM. Psilocin was found to exhibit a high affinity for 5-HT_1A_ receptors, with *K*_i_ = 49 nM (Blair et al., 2000). WAY-100635 entirely blocked the effect of psilocin on locomotor activity, which suggests that this effect is mediated by 5-HT_1A_ receptors (Halberstadt et al., 2011). Thus, the fact that psilocin acts through 5-HT_1A_ receptors may clarify why indoleamine psychedelics induced a decrease in locomotor activity in the mouse BPM.

Furthermore, we examined the possibility that 5-HT_2A_ receptors are involved in the behaviour of mice exposed to DOM by testing whether 5-HT_2A_ antagonists blocked the hyperactivity induced by phenylalkylamine. M100907 completely blocked the increase in locomotor activity produced by DOM. Consistent with our findings, Halberstadt *et al.* also reported that low doses of DOM and mescaline increase locomotor activity by activating the 5-HT_2A_ receptor, and high doses reduce activity in mice, whereas psilocin induces a profound suppression of locomotor activity (Halberstadt et al., 2011; Halberstadt and Geyer, 2011; Halberstadt et al., 2013). Given other findings regarding DOM, mescaline, and psilocin in the mouse BPM, we reasoned that the mouse BPM may be able to detect subtle behavioural differences between phenylalkylamine and indoleamine psychedelics.

To our knowledge, aside from horizontal locomotor activity, the behavioural response of mice to novelty typically manifests as vertical activity, namely, rearing behaviour. In contrast to locomotor activity, rearing is considered to reflect both exploratory activity and an emotional response (Gironi Carnevale et al., 1990). A defensive animal will likely spend more time along the perimeter, especially initially, indicating that the early behaviour in response to a walled environment may be dominated by supported rearing. As far as we know, our experiments are the first to demonstrate that rearing frequency followed an inverted U-shaped dose-response function, with low and moderate doses of DOM increasing rearing and high doses decreasing rearing. Nevertheless, DOM consistently reduced holepoking within the dose range. As shown in the current experiment, mescaline and psilocin both induced DOM-like effects on holepoking and rearing behaviours. In addition, a similar trend was observed regarding jumping mediated *via* 5-HT_2A_ receptors. There is convincing evidence that specific brain regions associated with defensive behaviours, such as the periaqueductal gray (PAG) and medial hypothalamus, are involved in both rearing and jumping, supporting the notion of rearing as a flight behaviour (Sandner et al., 1987; Silveira and Graeff, 1988; Silveira and Graeff, 1992). However, in some sites, jumps were reliably induced without an equal increase in rearing (Di Scala et al., 1984). It may be more appropriate to consider rearing as an orienting escape response compared to the explosive escape response of jumping. Notably, rearing and jumping are dissociable according to the test conditions.

Earlier research demonstrated that the effects of DOI on rearing behaviour were diminished in 5-HT_2A_ KO mice (Halberstadt et al., 2009). To validate our findings in the 5-HT_2A_ KO mice as being caused by the absence of the receptor, we also examined whether the DOM-induced increase in rearing could be blocked by a 5-HT_2A_ antagonist. First, the DOM-induced increase in rearing, an exploratory behaviour, was blocked by the 5-HT_2A_ antagonist M100907. However, DOM-induced changes in holepoking behaviours at the dose range tested were not blocked by M100907. Second, as a highly selective agonist of the 5-HT_2A_ receptor, 25CN-NBOH also exhibits classic hallucinogenic properties; 25CN-NBOH significantly increased rearing in our study, and this effect was blocked by M100907. Notably, lisuride, an ergoline derivative, is a structural analogue of LSD and shows a similar binding profile at monoamine receptors (Halberstadt and Geyer, 2018). Although lisuride has a high affinity for 5-HT_2A_ receptors (*K*_i_ = 12 nM), it does not produce hallucinogenic effects (Pieri et al., 1978; Egan et al., 1998; Benes et al., 2006). In the PPI, a cross-species behavioural paradigm, lisuride and LSD disrupted the PPI through distinct receptor mechanisms, providing additional support for the classification of lisuride as a nonhallucinogenic 5-HT_2A_ agonist (Halberstadt and Geyer, 2010). In our study, even 3.2 mg/kg lisuride failed to increase rearing behaviour. Although these findings do not completely exclude the possibility that the behavioural inactivity of lisuride is due to partial agonist activity, they confirm that lisuride does not induce rearing behaviour in mice. Moreover, TBG, a putatively nonhallucinogenic agonist of 5-HT_2A_ receptors, showed no effect on rearing behaviour. While TBG does not produce the head-twitch response of psychedelics in rodents (Cameron et al., 2021), only human clinical studies can ultimately confirm that it is nonhallucinogenic. Nevertheless, our work highlights that the DOM-induced increase in rearing behaviour at low to medium doses is predominantly mediated by 5-HT_2A_ receptors, which is distinct from the effects of the nonhallucinogenic 5-HT_2A_ agonist lisuride and the putatively nonhallucinogenic 5-HT_2A_ agonist TBG in the mouse BPM.

Similar to our findings, low doses of DOM markedly enhanced locomotor activity in ddN mice (Yamamoto and Ueki, 1975). In contrast to the current findings, those researchers discovered that DOM decreased rearing when administered at dosages above 0.5 mg/kg. However, Yamamoto and his colleagues assessed rearing behaviour in an open field before commencing the experiment, in which animals were matched based on two predrug sessions, with a delay between the initial testing phase and the onset of increased rearing. In our BPM study, mice were confined to the chamber for 60 min directly following DOM administration. Thus, rearing may increase in situations of uncertainty (such as when the desire to explore a particular environment is roughly equal to perceived danger) but reduced in situations where balance is polarized (e.g., when the environment is either very safe or very dangerous). Thus, it is likely that these disparate findings are the result of different study designs and that the duration of observations used by open-field studies with DOM is probably insufficient to detect a psychedelic-induced increase in rearing behaviour. There is also evidence that the IP administration of DOM, mescaline, and psilocin to C57BL/6J mice reduces exploratory holepoking and rearing (Halberstadt et al., 2011; Halberstadt and Geyer, 2011; Halberstadt et al., 2013). It is important to note, however, that Halberstadt and colleagues used different apparatuses to assess exploratory behaviour (Halberstadt et al., 2011). It is known that rearing behaviour is highly sensitive to the environmental context, including lighting conditions, noise, sex, and stress exposure (Sturman et al., 2018). Therefore, these factors will likely influence the experimental results.

Next, we determined whether the increase in rearing reflected fear-like behaviour. We determined three criteria based on previous studies: defensive behaviours (usually freezing) (Fendt et al., 2005), altered autonomic functions such as defecation (Ekman et al., 1983), and anxiety-like behaviour during the entire testing session (Blanchard and Blanchard, 1988). Innate fear is a basic and natural response that allows animals and humans to avoid danger. Emotions are triggered by a threat perceived through the senses, which normally initiates an immediate response, such as freezing, fleeing, or hiding, and thus plays an important role in survival (Ohman and Mineka, 2001). Notably, after administration of an effective dose, there was no significant difference in freezing evoked by DOM, mescaline, or psilocin. Several studies in animals and humans have shown that the autonomic nervous system is rapidly affected by fear (Ekman et al., 1983; Nestler et al., 2002). Consistent with prior findings, we observed that DOM, mescaline, and psilocin at the dose range tested all decreased defecation in mice. According to Dang *et al.*, the length of time spent in the central area is an indicator of anxiety-related behaviour (Dang et al., 2022). Anxiety is considered low if the length of time spent in the central area is high. The amount of time spent in the central area was comparable between the control group and psychedelic groups. Thus, our results show that the increase in rearing behaviour evoked by DOM and other psychedelics was inconsistent with fear because the behavioural changes induced by psychedelics did not meet all criteria for fear-like behaviour.

Many behavioural observations act as matrices of probabilistic variation and require a higher level of statistical analysis than mere descriptive statistics to evaluate all data in an integrated framework (Leighty et al., 2004). The ANOVA results indicated differences in the hallucinogenic group for all behavioural categories except for “jumping behaviour” induced by the lowest effective doses of mescaline, psilocin, and 25CN-NBOH. Thus, we cannot draw clear conclusions based only on the results of univariate statistical analysis methods because other links between behavioural parameters have not been clarified. We found that canonical discriminant Function 1 explained 94.8% of the variance and was statistically significant (*p* < 0.001). Among the seven behavioural parameters in this study, “Rearing” was strongly correlated with Function 1, whereas “Freezing” had a weak correlation, indicating that “Rearing” was the discriminant variable that contributed the most to group differences. In summary, a higher level of multiscale analysis of behavioural measures in animal models would be useful for further evaluating the efficacy of psychedelics.

Consistent with our pharmacological studies, exploration at the level of the individual animal occurs due to the competition of two motivational factors: “exploratory drive” and anxiety or fear (Montgomery, 1955). Based on this two-factor framework, the increase in exploratory behaviour may be attributed not only to greater exploratory motivation but also to ‘disinhibition’ caused by a reduction in anxiety or fear. In other studies, Lever et al. (2006) reported that subicular-accumbens neurons triggered rearing and other exploratory movements in response to a novel environment, leading to an increase in rearing behaviour. Inhibition of this signal interferes with basolateral amygdala-accumbens neurons, which in turn facilitates fear-related immobilization, ultimately resulting in a reduction in rearing behaviour (Burns et al., 1996; Mulder et al., 1998). Furthermore, it has been recently reported that apart from the striatum, the medial frontal cortex, an area associated with cognitive processes, motivation, and emotional states, plays a crucial role in promoting rearing (Bertolucci-D'Angio et al., 1990; Feenstra and Botterblom, 1996; Kolb, 1984). These studies reported lower serotonin levels in the tissues of mice exhibiting increased rearing, along with a trend towards lower noradrenaline levels. The neurochemical pattern of lower 5-hydroxytryptamine (5-HT) content in the frontal cortex and higher dopamine (DA) content in the striatum is consistent with the psychobiological model proposed by Zuckerman, who attributed high sensation seeking to an overactive dopaminergic system combined with mildly-responsive serotonergic and noradrenergic systems (Zuckerman, 1993; Zuckerman, 1996). In addition, microdialysis studies have shown that both 5-HT_2A_ and 5-HT_2C_ receptors regulate cortical dopamine efflux, but in opposite directions (Huang et al., 2011). It has been indicated that 5-HT_2C_ receptors tonically inhibit frontal cortical dopaminergic and adrenergic transmission (Millan et al., 1998). In contrast to the tonic and inhibitory effects of 5-HT_2C_ receptors, activation of 5-HT_2A_ receptors increased DA and norepinephrine levels but not 5-HT levels in the frontal cortex dialysate (Gobert and Millan, 1999). Pehek et al. (2006) suggested that this effect may be mediated by the activation of glutamatergic projections from corticotegmental DA neurons to mesocortical DA neurons. Thus, further studies are required to illuminate the possible relationships between these transmitters in the frontal cortex.

Hallucinogenic and nonhallucinogenic 5-HT_2A_ agonists differ in the neurobehavioural responses elicited according to the distinct signalling responses evoked by different ligands. Gonzalez-Maeso et al. (2007) reported that LSD and lisuride both act on 5-HT_2A_ receptors expressed by cortex neurons to regulate phospholipase C (PLC), while LSD specifically activates pertussis toxin- (PTX)- sensitive G_i/o_ proteins and Src. Moreover, immediate early gene c-Fos induction followed the activation of a G_q_-dependent pathway, which was activated by both hallucinogenic and nonhallucinogenic 5-HT_2A_ agonists. In contrast, early growth response-2 (Egr-2) induction is a downstream event of a G_i/o_-dependent pathway, which is selectively activated by psychedelics (González-Maeso et al., 2003; Gonzalez-Maeso et al., 2007; González-Maeso et al., 2008). Similarly, the DOI-induced phosphorylation of signalling molecules, namely, pCREB, pERK, pCaMKII, pPLC, and pPKC, as well as the production of IP3 and DAG, was substantially greater in magnitude than that evoked by lisuride (Banerjee and Vaidya, 2020). Samah *et al.* found biased phosphorylation of 5-HT_2A_ receptors at Ser^280^ in response to hallucinogenic *versus* nonhallucinogenic agonists (Karaki et al., 2014). The administration of DOI but not lisuride to mice enhanced the phosphorylation of Ser^280^ in the prefrontal cortex, reducing receptor desensitization by non-hallucinogenic agonists. Taken together, these findings suggest that differences in signalling responses induced by hallucinogenic and nonhallucinogenic 5-HT_2A_ agonists lead to differences in behavioural, electrophysiological, and transcriptional responses evoked by distinct ligands of 5-HT_2A_ receptors.

Several limitations of the present study need to be addressed by further investigations. Although the mouse BPM may be useful for the detection of subtle behavioural differences between phenylalkylamine and indoleamine psychedelics, the mechanisms underlying their psychological effects remain opaque at the cellular, molecular, and circuit levels. A critical step towards understanding the molecular mechanisms of psychedelics will be to develop paradigms that can distinguish between the behavioural effects of psychedelics and nonhallucinogenic psychedelic analogues. Future research should incorporate a variety of tools (e.g., imaging, electrophysiology, and behaviour) to better understand how hallucinogenic effects are generated at the molecular and circuit levels.

## Conclusion

In conclusion, the present findings indicate that the increase in rearing behaviour induced by phenylalkylamine psychedelics such as DOM is mediated by 5-HT_2A_ receptors. Thus, it is possible to relate other behavioural effects of phenylalkylamine psychedelics to their tendency to modulate rearing behaviour. We anticipate that these findings will accelerate efforts to identify behavioural parameters that can be utilized to assess the effects of psychedelics in various animal models and shed light on a variety of neuropsychiatric disorders.

## Data Availability

The original contributions presented in the study are included in the article, further inquiries can be directed to the corresponding authors.
